# Mitochondrial DNA Haplogroup Analysis Reveals no Association between the Common Genetic Lineages and Prostate Cancer in the Korean Population

**DOI:** 10.1371/journal.pone.0002211

**Published:** 2008-05-21

**Authors:** Wook Kim, Tag-Keun Yoo, Dong-Jik Shin, Hyun-Wook Rho, Han-Jun Jin, Eun-Tak Kim, Yoon-Sun Bae

**Affiliations:** 1 Department of Biological Sciences, Dankook University, Cheonan, Korea; 2 Department of Urology, Eulji Medical Center, Eulji University School of Medicine, Seoul, Korea; 3 Cardiovascular Genome Center, Yonsei University College of Medicine, Seoul, Korea; 4 Department of Urology, Eulji University School of Medicine, Daejeon, Korea; University of Montreal, Canada

## Abstract

Mitochondrial DNA (mtDNA) variation has recently been suggested to have an association with various cancers, including prostate cancer risk, in human populations. Since mtDNA is haploid and lacks recombination, specific mutations in the mtDNA genome associated with human diseases arise and remain in particular genetic backgrounds referred to as haplogroups. To assess the possible contribution of mtDNA haplogroup-specific mutations to the occurrence of prostate cancer, we have therefore performed a population-based study of a prostate cancer cases and corresponding controls from the Korean population. No statistically significant difference in the distribution of mtDNA haplogroup frequencies was observed between the case and control groups of Koreans. Thus, our data imply that specific mtDNA mutations/lineages did not appear to have a significant effect on a predisposition to prostate cancer in the Korean population, although larger sample sizes are necessary to validate our results.

## Introduction

Prostate cancer is one of the most common cancers and the second leading cause of cancer mortality in Caucasian men, but its incidence varies considerably between human populations [1]. Based on a recent survey, an approximately 1.6-fold greater incidence of prostate cancer was diagnosed and a 2.4-fold greater mortality reported in African-American men compared to European-American men [2]. In addition, Asian populations have shown a general trend of rising incidence of the cancer after adopting westernized lifestyles, although the incidence is still lower in Asia than Western countries [3], [4]. It appears that multiple variables including ethnic origins, environmental, and genetic factors are likely linked to prostate cancer [5]–[8].

Mitochondria have been implicated in malignancy and cancer biology, explained by their essential role in the generation of ATP and for regulating apoptosis [9], [10]. Recent studies suggest that mitochondrial DNA (mtDNA) mutations may play an important role in prostate carcinogenesis. For instance, a transmitochondrial cybrid experiment demonstrated that the resulting mutant cybrids appeared to generate tumors that were seven times larger than the wild-type cybrids, whereas the wild-type cybrids barely grew in the mice [7]. Jessie et al. [11] found that the average number of mtDNA deletions in the malignant prostate of patients increased with age. In addition, somatic mtDNA mutations that occur well before changes in tissue histopathology indicative of prostate cancer are highly informative about the oncogenesis of the disease [12].

Since mtDNA is haploid and lacks recombination, specific mutations in the mtDNA genome leading to human diseases arise in particular genetic backgrounds referred to as haplogroups [13]. Human populations usually carry several mtDNA haplogroups defined by unique sets of mtDNA polymorphisms, reflecting mutations accumulated by a discrete maternal lineage [14], [15], but the sets and their frequencies differ between populations. Thus, haplogroup association studies have been used to assess the role of mtDNA variants in various complex diseases. Recently, Booker et al. [8] noted that the inheritance of mtDNA haplogroup U was associated with an approximately 2-fold increased risk of prostate cancer and 2.5-fold increased risk of renal cancer in North American individuals with European ancestry. Such a finding needs to be investigated in an independent population to determine whether a causal role of mtDNA haplogroups in the cancer can also be detected elsewhere, for example in the different maternal lineages in east Asia.

To assess the possible contribution of mtDNA haplogroup-specific mutations to the occurrence of prostate cancer, we have therefore investigated the association between common mtDNA lineages and a predisposition to the cancer in the Korean population by examining 139 prostate cancer cases and 122 corresponding controls.

## Methods

### Patients and controls

We analyzed a total of 139 Korean prostate cancer patients, who were recruited for the study from the urology department of the Eulji University School of Medicine in Seoul and Daejeon, Korea. The DNA samples included subsets of the samples examined by Kim et al. [16]. Histological classification of prostate cancer was determined according to the World Health Organization (WHO) recommendations and the Gleason pattern (Table 1). Prostate cancer tissue specimens from all of the patients were collected from frozen samples. In addition, a total of 122 Korean men who had been diagnosed as free of prostate cancer by the Eulji University hospital in Seoul and Daejeon, Korea were recruited as normal controls. These subjects were selected at random (and therefore likely to be unrelated) from the same geographical area as the cases. This study was approved by the Ethics Committee and institutional review boards of Eulji Medical Center of the Eulji University School of Medicine in Seoul, and separate written informed consent was obtained for screening and for enrollment from all participants.

**Table 1:** Clinicopathological characteristics of Korean prostate cancer patients and control groups surveyed here.

Characteristic	Category	Prostate Cancer Patient (n = 139)	Control (n = 122)
Age (year)	≤55	5 (3.60%)	39 (31.97%)
	56–60	11 (7.91%)	20 (16.39%)
	61–65	25 (17.99%)	33 (27.05%)
	>65	98 (70.50%)	30 (24.59%)
		[70.6±8.3]*	[59.6±10.8]*
PSA (mg/ml)	≤4.0	15 (10.79%)	113 (92.62%)
	4.1–10.0	41 (29.50%)	3 (2.46%)
	10.1–20.0	24 (17.27%)	3 (2.46%)
	>20.0	59 (42.45%)	-
	Not available	-	3 (2.46%)
Gleason score	2–6	47 (33.81%)	-
	7	35 (25.18%)	-
	8–10	56 (40.29%)	-
	Not available	1 (0.72%)	-

* Mean age±SD.

### DNA extraction and genotyping

Genomic DNAs for patients and controls were extracted from peripheral blood leukocytes using standard protocol [17]. Screening for mtDNA haplogroups D, D4a, D4b, D4, G, M7, M7a, M7b, M8, M8a, M, N, N9, Y, A, B, and F in Koreans (Table 2) was performed using the multiplex amplified product length polymorphism (APLP) method [18]. Each PCR reaction was performed in a total volume of 20 µl containing 25 ng of genomic DNA, 0.2 mM dNTPs, 1.5 mM MgCl_2_, 1× PCR buffer (Applied Biosystems, Foster city, CA, USA), 1.0 U ampliTaq Gold™ DNA polymerase (Applied Biosystems, Foster City, CA, USA) and appropriate concentration of primers. Primers and concentrations were described in Umetsu et al. [18]. The PCR amplification was carried out using a GeneAmp® PCR system 9700 thermal cycler (Applied Biosystems, Foster City, CA, USA) under the conditions: 95°C for 15 min, 32 cycles of 94°C for 10 sec, 52°C for 10 sec, 72°C 5 sec, and a final extension at 72°C for 3 min. The band patterns of the alleles were evaluated on 10% native PAGE gels and visualized by silver staining. Haplogroups D5, G2, C, B5 and F1 (Table 2) were determined by PCR-RFLP method [19]. The resulting restriction fragments were resolved by electrophoresis in 1.5% QA-Agarose™ agarose (Q-BioGene, OH, USA) gels, visualized by ethidium bromide staining. The nomenclature of the mtDNA haplogroups followed previous reports [20], [21]. All samples were classified on the basis of the criteria shown in Table 2.

**Table 2:** Polymorphic multiplex APLP and restriction variants determining mtDNA haplogroups in this study.

Haplogroup	Key multiplex APLP and restriction* variants
M*	10400T
D*	5178A 10400T
D4	3010A 5178A 10400T
D4a	3010A 5178A 10400T 14979C
D4b	3010A 5178A 8020A 10400T
D5	5178A -10394*Dde*I/-10397*Alu*I 10400T
G*	4833G 10400T
G2	4833G *-7598Hha*I 10400T
M7*	6455T 10400T
M7a	4386C 6455T 10400T
M7b	6455T 10400T 12811C
M8*	10400T 15487T
M8a	8684T 10400T 15487T
C	10400T -13259*Hinc*II/+13262*Alu*I 15487T
N*	10398A/10873T
N9*	5417A 10398A/10873T
Y	5417A 10398A/10873T 14178C
A	1736G 10398A/10873T
B*	9-bp deletion 10398A/10873T
B5	9-bp deletion +10394*Dde*I 10398A/10873T
F*	3970T 10398A/10873T
F1	3970T 10398A/10873T -12406*Hpa*I/*Hinc*II

* Sites are numbered from the first nucleotide of the recognition sequence. A plus sign (+) indicates the presence of a restriction site, and a minus sign (−) indicates the absence of restriction site.

### Data analyses

Mitochondrial DNA haplogroup frequencies were calculated by counting from the observed phenotypes. To test for significant population differentiation between all pairs of samples for the prostate cases and the control groups, we used an Exact Test implemented in the Arlequin package version 2.0 [22]. The significance level of the test was applied with a probability of <0.05 as cutoff point. In addition, a test of proportion and odds ratios (OR) with 95% confidence intervals (CI), and a Fisher’s Exact Test on a 2×2 table were calculated using the statistical software program (http://www.quantitativeskills.com/sisa/).

## Results and Discussion

**Figure 1:**
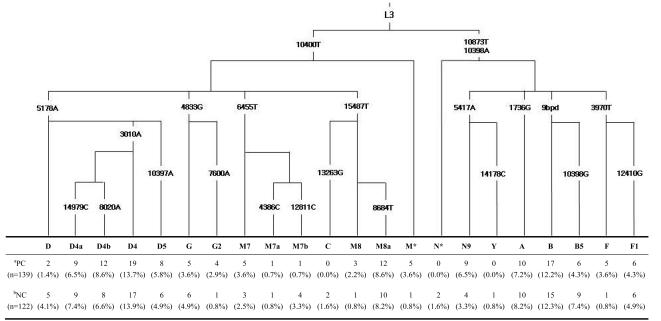
Mitochondrial DNA haplogroup distribution in prostate cancer cases and controls in the Korean population. The parsimonious tree on the top shows the evolutionary relationship of 22 haplogroups; capital letter-number codes denote haplogroups, all mutations are indicated by the substituted nucleotides after the number. ^a^Prostate cancer; ^b^Normal control; Exact test *P* value (s.e.) = 0.6064±(0.0374).

We analyzed 22 mtDNA haplogroups using APLP and PCR-RFLP methods in the cancer and control samples, most of which are the common set of haplogroups in east Asia. The haplogroup-based phylogenetic analysis used here enhances mtDNA database quality, thus minimizing noise that would hinder the interpretation of the variation in our samples [23]. Frequency distributions of the prevalence of mtDNA haplogroups and subhaplogroups in Koreans are listed in Figure 1. The Korean population surveyed here is characterized by a high frequency of haplogroup D4 lineages (and its sublineages) in both groups of prostate cancer patients (28.8%) and normal controls (27.9%) (Figure 1). In total, thus, mtDNA haplogroups D and their subhaplogroups (D4a, D4b, D4, and D5) were found to be the most prevalent maternal lineages in both samples of prostate cancer patients (36.0%) and normal controls (36.9%), and are widespread in northeast Asia. In addition, southeast Asian-prevalent mtDNA lineages of (sub)haplogroups B, M7, and F are also found at moderate frequencies in the population. This result is consistent with previous mtDNA and Y-chromosomal DNA surveys, showing that the Koreans possess lineages from both the southern and northern haplogroup complexes of east Asia [20], .

Haplogroup U, reported by Booker et al. [8] to be over-represented in prostate cancer patients in North American individuals, was absent from our sample, which made it impossible to assess the correlation between this lineage and cancer cases. Based on our result, there were no statistically significant differences in the distribution of mtDNA haplogroup frequencies between all pairs of samples for the case and control groups (Figure 1). In addition, each haplogroup was first tested separately and then phylogenetically-combined haplogroups were tested using OR and their 95% CI, and a Fisher’s exact test (Table 3). We found no association between the cancer and the control groups for each haplogroup. When comparing the distribution of phylogenetically-combined haplogroups in patients to that in the control group, there were again no significant differences between cancer groups and the control distribution for haplogroups D, G, M7, M8, N9, B, and F (Table 3). Thus, our data imply that specific mtDNA mutations/lineages did not appear to have a significant effect on a predisposition to prostate cancer in the Korean population. However, the sample size of this survey may not provide adequate power to detect associations with the large number of haplogroups present in the sample (Figure 1). Thus, further studies with larger sample sizes are necessary to validate our results, because even association with fairly common haplogroups and large effects may be missed with the limited sample sizes.

**Table 3:** Analysis of association between prostate cancer risk and the mtDNA haplogroups in this study.

	Odds Ratio	95% CI	P value*
D	0.3416	0.0651<OR<1.7936	0.13
D4a	0.8692	0.3336<OR<2.2650	0.18
D4b	1.3465	0.5314<OR<3.4115	0.15
D4	0.9779	0.4833<OR<1.9788	0.14
D5	1.1807	0.3979<OR<3.5033	0.21
G	0.7214	0.2146<OR<2.4255	0.21
G2	3.5852	0.3953<OR<32.5199	0.19
M7	1.4801	0.3463<OR<6.3257	0.25
M7a	0.8768	0.0543<OR<14.1699	0.50
M7b	0.2138	0.0236<OR<1.9391	0.13
C	-	-	0.22
M8	2.6691	0.2740<OR<26.0021	0.28
M8a	1.0583	0.4403<OR<2.5433	0.18
M	4.5149	0.5201<OR<39.1925	0.12
N	-	-	0.22
N9	2.0423	0.6127<OR<6.8071	0.12
Y	-	-	0.47
A	0.8682	0.3487<OR<2.1620	0.18
B	0.994	0.4736<OR<2.0860	0.15
B5	0.5664	0.1956<OR<1.6398	0.12
F	4.5149	0.5201<OR<39.1925	0.12
F1	0.8722	0.2738<OR<2.7785	0.22
D group	0.9613	0.5800<OR<1.5932	0.10
G group	1.1374	0.4105<OR<3.1513	0.20
M7 group	0.7557	0.2658<OR<2.1485	0.18
M8 group	1.2207	0.5381<OR<2.7691	0.15
N group	1.3385	0.4624<OR<3.8746	0.18
B group	0.8096	0.4303<OR<1.5232	0.10
F group	1.4118	0.5296<OR<3.7638	0.15
M total	1.0525	0.6390<OR<1.7338	0.10
N total	0.9501	0.5768<OR<1.5650	0.10

Abbreviations: CI, confidence interval; OR, odds ratio.

Each haplogroup (hg) was first tested separately and then each phylogenetically-combined group was also tested.

* The Fisher exact test of proportions.

Recently, Kim et al. [16] also found no association between Y chromosome haplogroups and the relative risk of prostate cancer, which contrasts with previous findings in the Japanese-American population [25]. Nuclear gene mutations have also been linked to mitochondrial diseases because most proteins involved in mtDNA maintenance are nuclear-encoded [15]. Thus, further works on nuclear gene disorders that contribute to the onset of the mitochondrial diseases need to be investigated for better understanding of the pathogenesis of the disease.

Age and family history are important risk factors for prostate cancer, together with geographical origin [26]. We cannot rule out the possibility of the effect of age difference between the case and control samples, since it is not clear whether some controls may subsequently go on to develop prostate cancer. In fact, it should be noted that the mean age of controls (59.6±10.8) is less than that of cancer patients (70.6±8.3) (Table 1). Nevertheless, this effect seems likely to be small. Recent surveys from Asia (e.g., Japan, Singapore and Korea) have shown a general trend of a rising incidence of prostate cancer [3]. The changing demography of prostate cancer in Asia may better be explained by environmental factors. This possibility is supported by the observation that many Asian countries may be losing their protective dietary habits and acquiring high-risk ones by adopting westernized lifestyles [4], [16]. It leads us to conclude that multiple variables including ethnic background, environmental, and genetic factors are likely linked to prostate cancer.

Thus, further surveys on analyses of this association using additional genetic markers and larger diverse samples could help to evaluate the joint actions of genetic background and environmental factors for the fuller understanding of the etiology of prostate cancer.
